# Effect of environmental factors on the germination and emergence of *Salvia verbenaca* L. cultivars (*verbenaca* and *vernalis*): An invasive species in semi-arid and arid rangeland regions

**DOI:** 10.1371/journal.pone.0194319

**Published:** 2018-03-22

**Authors:** Muhammad Mansoor Javaid, Singarayer Florentine, Hafiz Haider Ali, Sandra Weller

**Affiliations:** 1 Department of Agronomy, University College of Agriculture, University of Sargodha, Sargodha, Pakistan; 2 Centre for Environmental Management, Faculty of Science and Technology, Federation University Australia, Mt Helen, Ballarat, Vic, Australia; College of Agricultural Sciences, UNITED STATES

## Abstract

*Salvia verbenaca* (wild sage) is a commonly cultivated herbal medicine plant, which is native to the Mediterranean climate regions of Europe, Africa, Asia and the Middle East. However, it has become an invasive species in semi-arid and arid regions of southern Australia. Two varieties are present in this region, var. *verbenaca* and var. *vernalis*, each of which can be distinguished by differences in morphology and flowering period. Following trials to determine the optimum temperate regime for germination and response to light and dark, seeds of both varieties were tested for their response to variations in pH, moisture stress, salinity, and burial depth. The temperature and light trial was carried out using three different temperature regimes; 30/20°C, 25/15°C and 20/12°C, and two light regimes; 12 hours light/12 hours dark and 24 hours dark, with var. *vernalis* responding to relatively higher temperatures than var. *verbenaca*. The germination rate of neither species was significantly inhibited by complete darkness when compared to rates under periodic light exposure. Both varieties germinated at near optimum rates strongly to very strongly in all pH buffer solutions, from pH 5 to pH 10, but they responded most strongly at neutral pH. Var. *vernalis* showed slightly more tolerance to reduced moisture availability, moderate to strong salinity, and burial depth, compared to var. *verbenaca*. However, even a fairly shallow burial depth of 2 cm completely inhibited germination of both varieties. Thus, in circumstances where both varieties are present in a soil seedbank, var. *vernalis* could be expected to establish in more challenging conditions, where moisture is limited and salinity is ‘moderate to high’, implying that it is a more serious threat for invasive weed in conditions where crop plants are already challenged.

## Introduction

*Salvia verbenaca* L. (wild sage) (F. Lamiaceae) is native to western and southern Europe, the Middle East, North Africa and western Asia [1]. As is common practice in several parts of the world for other *Salvia* species (e.g., *S*. *officinalis*, *S*. *fruticosa*, *S*. *lavandulaefolia* and *S*. *sclarea*), *S*. *verbenaca* is deliberately cultivated to (i) obtain dried leaves, which are used as raw material in herbal medicines and (ii) provide essential oils which are extracted from the plant [2]. More recently, however, it has inadvertently become an invasive environmental weed of semi-arid and arid regions of Australia [3]. Two varieties of *S*. *verbenaca*, var. *verbenaca* and var. *vernalis*, are present in New South Wales, Victoria, South Australia and Tasmania [4]. These two varieties can be distinguished easily at seedling or lateral growth stages, as follows. The lower leaves of var. *verbenaca* are ovate in outline, shallowly lobed to subentire, glabrescent, and not or only sparsely glandular beneath. The calyx is often purplish and it flowers from June to November [5]. By contrast, var. *vernalis* has lower leaves that are oblong in outline, pinnatifid, shortly hirsute and strongly glandular beneath. The calyx is green and it flowers from March to October [5]. Additionally, var. v*ernalis* plants are somewhat more erect than those of var. *verbenaca*, which appears to be more shrub-like in appearance. As has been observed with other introduced weed species, both varieties may vary in their growth habitat and respond differently to different environments, which makes knowledge of their habits a priority for sensible management and control.

Environmental factors, such as temperature, light, pH, osmotic and salt stress affect the germination or emergence of weeds [6], [7], [8], [9]. Some weeds have a narrow tolerance range, while others are able to germinate over a wide range of temperatures [10], [9]. Water stress may delay or prevent the germination of seeds [11], and the effect of drought on germination may vary according to weed species or between different populations of same weed [12], [13]. Salinity may affect seed germination according to species, genotypes and environmental conditions [14] and germination may be reduced when salt accumulation exceeds a threshold level [15], [16]. Soil pH also affects weed seed germination, although some weeds can tolerate a wide range of pH levels [6]. Seed burial depth is an important factor that may influence the seed germination or seedling emergence, by affecting light, moisture and temperature [17].

Because germination and seedling emergence are the most critical phases in plant development [18], as indicated earlier, any information that can be obtained about optimum conditions for germination and establishment of a weed species may assist with designing efficient measures for its control. Consequently, to measure the effects of temperature and light, pH, osmotic stress and salinity on seed germination, and the effect of burial depth on seed germination and emergence, the seeds of both *Salvia* varieties were subjected to a series of germination trials. Establishing the ecological context of seed germination and the emergence of each variety can help to characterize the respective germination niches developed within their natural habitat, and therefore give information on which management actions are likely to assist with control of each variety in an agricultural or conservation setting.

## Materials and methods

### Seed source

Mature and seeds of both varieties were collected at two locations. *S*. *verbenaca* var. *verbenaca* seeds were collected from approximately 75 mature plants during November 2014 from Birchip in north-western Victoria (35°58.59.94°S, 142°54.52.41°E). The semi-arid climate in this region has hot, dry summers and cool winters [19], with an annual average rainfall of 300–400 mm and an average annual evaporation of between 1200 to 1400 mm [20]. Seeds of *S*. *verbenaca* var. *vernalis* were collected during November 2014 from more than 100 plants at different locations in the Federation University’s Nanya Research Station (33.12.33°S, 141.19.09°E) in New South Wales. This property is located in the arid-zone of Australia. The climate is cool-arid with annual average annual rainfall of between 200–300 mm and annual evaporation of between 2000 to 2400 mm [20]. Authors would like to point out state clearly that no specific permissions were required for the seed collected from Birchip in north-western Victoria (35°58.59.94°S, 142°54.52.41°E), and Federation University’s Nanya Research Station (33.12.33°S, 141.19.09°E) in New South Wales. This is mainly because seeds were collected along the road side at Birchip and Nanya Research Station belongs to Federation University where Singarayer Florentine is currently employed. Given that that this is an invasive species no specific permission required. We can also confirm that the field studies did not involve endangered or protected species.

The seeds from both locations were contained within seed capsules at the time of collection. Seed capsules were placed in paper bags and transported to the seed ecology lab of the Federation University Australia at the Mt Helen campus, where the seeds were separated from the capsules, cleaned, and air-dried for seven days at room temperature. The seeds were stored in labelled, air-tight glass bottles until used in the germination experiments.

The seed weight of each variety was measured. Ten lots of 100 seeds were weighed to four decimal places (Sartorius GMBH Gottingen, Type B120S) and the mean seed weight and standard deviation calculated. The var. *verbenaca* seeds weighed 2.40 ± 0.045 mg and the var. *vernalis* seeds weighed 1.75 ± 0.014 mg.

### General seed germination protocols

Initial germination attempts indicated that the seeds of both varieties were dormant. To overcome dormancy, seeds were treated with a 100-ppm solution of gibberellic acid, in which they were soaked for 12 hours prior to additional germination treatments being applied.

In each germination trial there were six replicates of 20 seeds, three replicates for the alternating light regime, 12 hours light/12 hours dark, and three for the complete darkness regime of 24 hours dark. For the latter, to ensure darkness the Petri dishes were wrapped with aluminium foil. Subsequently, to avoid any exposure to white light during sample treatment which may inadvertently stimulate germination during the assessment of seed germination, these Petri dishes were opened under a green safe light. Before the start of each experiment, seeds were surface sterilized by soaking in 1% sodium hypochlorite (NaClO) for five minutes, then rinsed five times with distilled water.

Following surface sterilization, seeds were placed evenly into a 9 cm diameter Petri dish lined with Whatman^®^ No. 11 filter paper. A 5 ml volume of either sterilized distilled water or the relevant treatment solution was added to each Petri dish, which was subsequently sealed with Para-film^®^ to prevent moisture loss. Petri dishes were placed into an incubator (Thermoline Scientific, Temperature and humidity Cabinet, Model: TRISLA-495-1-SD, Vol 240, Australia), with cool-white fluorescent lamps that produced a photosynthetic photon flux of 40 μmol m^-2^ s^1^. Germination was assessed daily and seeds with a radicle length of 2 mm were counted as germinated.

### Effect of temperature and photoperiod

To establish the optimum temperature range for germination of *S*. *verbenaca*, seeds of each variety were incubated under fluctuating day/night temperatures of 30/20°C, 20/15°C, and 25/12°C. These temperature ranges were selected based on temperature variation during the growing season of *S*. *verbenaca* in the regions from which the seeds were sourced [20]. The effect of photoperiod was also investigated with the two different light regimes, of alternating light and complete darkness, as described previously.

Based on the finding of this trial, all subsequent experiments were conducted at 30/20°C day/night temperature with a 12 hours photoperiod, since this was the optimum germination condition for both species.

### Effect of osmotic and salt stress

Seeds of each variety were tested for germination in aqueous solutions of varying osmotic potential, -0.05, -0.1, -0.2, -0.4 and -0.6 MPa. Each solution was prepared by dissolving the appropriate weight of polyethylene glycol (PEG 8000; Sigma-Aldrich Co., 3050, Spruce St., MO 63130) in distilled water [21]. To assess the germination ability of each *S*. *verbenaca* variety under salt stress, seeds were germinated in sodium chloride (NaCl) solutions of varying concentrations that could be typical of salt-affected regions. These concentrations were 50, 100, 150, 200 and 250 mM. For each of these trials, a treatment with sterilized distilled water was included as a control.

### Effect of pH

To examine the effect of pH on germination, seeds were germinated in six different buffer solutions, pH 5, 6, 7, 8, 9 and 10. These solutions were prepared according to the method described by Chachalis and Ready (2000) [22]. The pH 5 and 6 buffers were prepared from a 2 mM solution of MES [2-(N-morpholino) ethanesulfonic acid] and adjusted to each respective pH value with 1N hydrochloric acid (HCl). The pH 7 and 8 buffers were prepared from a 2 mM solution of HEPES [N-(2-hydroxy-methyl) piperazine-N-(2-ethanesulfonicacid)] and the pH 9 and 10 buffers from a 2-mM TRICINE [N Tris (hydroxymethyl) methylglycine]. These four buffers were adjusted to each respective pH value with 1 N NaOH. Unbuffered deionised water (pH 6.2) was used as a control treatment.

### Effect of seeding depth

The effect of burial depth on seedling emergence of both varieties was studied in a glasshouse experiment, conducted at the Mt Helen campus of Federation University. Sandy loam soil was collected from the areas where the seeds were sourced, and autoclaved to kill any existing seeds or propagules. For both varieties, two trials, each with three replicates, were conducted using plastic pots of 15 cm diameter. In each replicate, 20 seeds were placed either on the soil surface (0 cm, control) or buried in the soil at varying depths within the pot (0.5, 1.0, 2.0, 3.0, 4.0 and 5.0 cm). Throughout the experiment, the temperature of the glass house was maintained at 27± 2°C during the day and 23 ±3°C at night. To maintain adequate soil moisture, the pots were watered daily. Seedlings were considered to have emerged when cotyledons were visible at the soil surface.

### Statistical analysis

Each experiment was arranged in a completely randomised design, with three replications repeated once, making six replicates overall for both varieties in each experiment. Data were combined from all replicates and subjected to analysis of variance (ANOVA). Two-way ANOVA was used to assess the effect of light and photoperiod on germination parameters of each *S*. *verbenaca* variety. The significant differences among treatment means were identified by Tukey’s Honestly Significant Difference (HSD) at *p* ≤ 0.05 [23].

Germination or emergence percentage data of each *S*. *verbenaca* variety obtained from the experiments regarding osmotic stress, salt stress and burial depth, were subjected to non-linear regression analysis. Germination (%) values at different concentrations of osmotic potential and NaCl were fitted to a functional three-parameter logistic model using Sigma Plot 2008 (version 11.0). The model fitted was:

$$G(\%)=\frac{G_{\max}}{1+{\left(\frac{x}{x_{50}}\right)}^{g}}$$ (1)

where *G* is the total germination (%) at concentration *x*, *G*_*max*_ is the maximum germination (%), *x*_*50*_ is the osmotic potential or NaCl concentration for 50% inhibition of the maximum germination and *g* indicates the slope.

A three-parameter logistic model:

$$E(\%)=\frac{E_{\max}}{1+{\left(\frac{x}{x_{50}}\right)}^{e}}$$ (2)

was fitted to the seedling emergence (%) of each variety obtained at different burial depths of 0 to 5 cm, where *E* is the total seedling emergence (%) at burial depth *x*, *E*_*max*_ is the maximum seedling emergence (%), *x*_*50*_ is the burial depth for 50% inhibition of the maximum seedling emergence and *e* indicates the slope.

The time taken to 50% germination or emergence (*T*_*50*_ or *E*_*50*_) was calculated according to the formula described by Coolbear et al. (1984) [24]:

$$T_{50}\;or\;E_{50}=t_{i}\frac{\left(\frac{N}{2}-n_{i})(t_{j}-t_{i}\right)}{\left(n_{j}-n_{i}\right)}$$ (3)

where *N* is the final number of germinated or emerged seed, and *n*_*j*_ and *n*_*i*_ are the cumulative number of seed germinated by adjacent counts at times *t*_*j*_ (day) and *t*_*i*_, (day), respectively, when *n*_*i*_ < *N*/*2* < *n*_*j*._

Mean germination or emergence time (*MGT* or *MET*), which is a measure of the speed of germination or emergence [25], was calculated after Ellis and Roberts (1981) [26]:

$$MGTorMET=\frac{\sum Dn}{\sum n}$$ (4)

where *n* is the number of seeds that had germinated on day *D* and *Dn* is the number of days counted from the beginning of germination experiment. The germination or emergence index (*GI* or *EI*), which is a measure for percentage and rate of germination (Kader 2005), was calculated as described by the Association of Official Seed Analysis (1993) [27] using the following formula:

$$GI\;or\;EI=\frac{No\;of\;germinated\;or\;emerged\;seedlings}{Days\;of\;first\;count}+\cdots \cdot +\frac{No\;of\;gerimnated\;or\;emerged\;seedlings}{Days\;of\;final\;count}$$ (5)

## Results and discussion

### Effect of light and temperature

**Fig 1:**
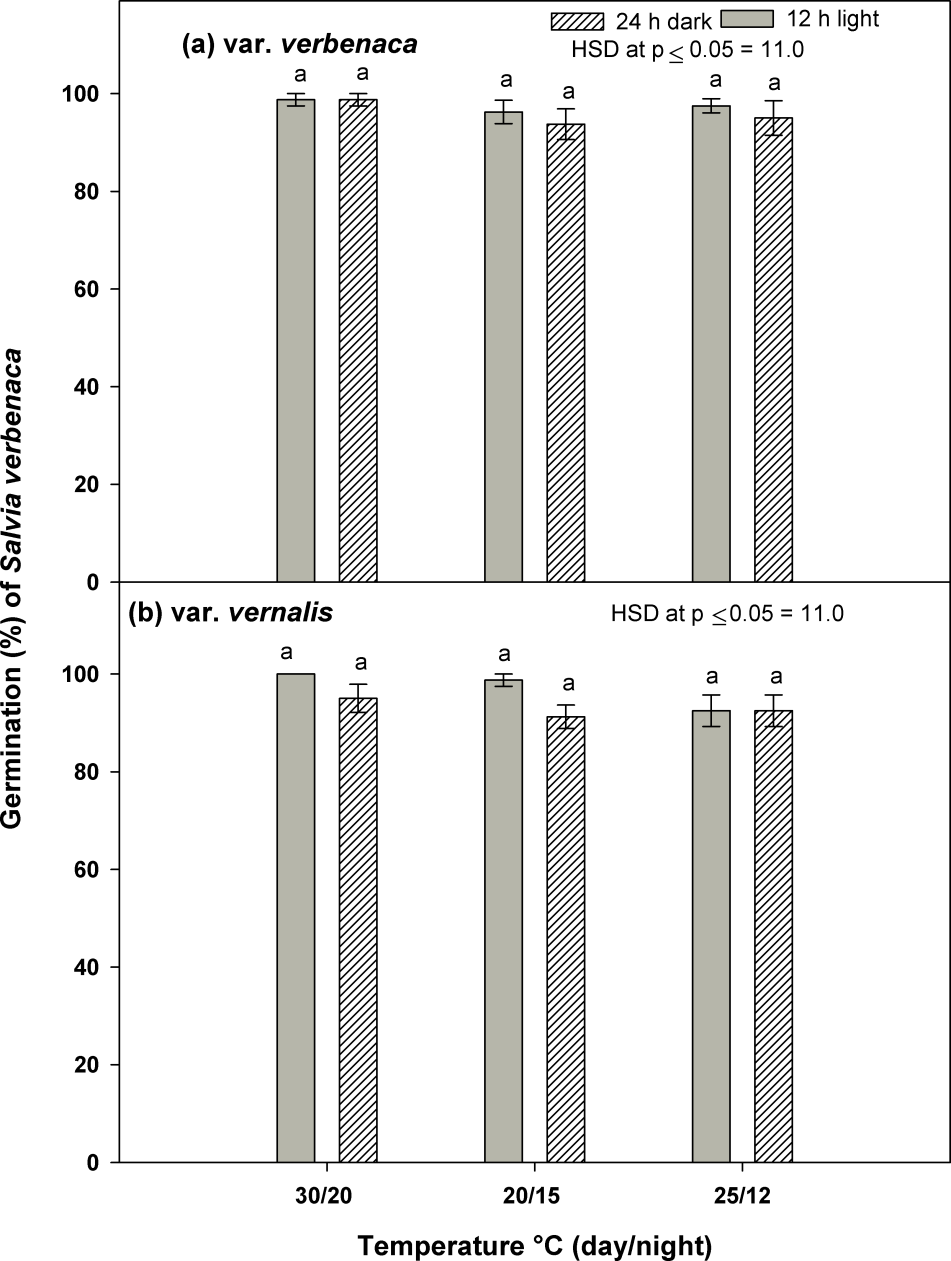
**Effect of temperature and photoperiod on germination of *S*. *verbenaca* varieties (a) *verbenaca* and (b) *vernalis*.** Nails on the vertical bars represent standard error of the means.

Overall, the effects of the light regime (alternating light or complete darkness) and day/night temperature (30/20°C, 20/15°C and 25/12°C) on the germination of each *S*. *verbenaca* variety did not vary significantly according to variety (Fig 1A and 1B). Under the alternating light regime, both species germinated very strongly, with maximum germination of var. *verbenaca* (96%) at 30/20°C and 20/15°C (Fig 1A) and maximum germination of var. *vernalis* (97%) at 30/20°C (Fig 1B). In the temperature range 25/12°C, and under the same light conditions, germination of both varieties was reduced only very slightly, to 93%. Under the complete darkness regime, germination of both varieties was only slightly reduced in all three temperature ranges, compared to the alternating light regime (Fig 1A and 1B).

In the highest temperature range, 30/20°C, germination of var. *verbenaca* was slightly slower to commence than var. *vernalis* (Tables 1 and 2). However, there was no difference in the time to commence germination in var. *verbenaca* in either light regime (3.0 days for both), and only a slight delay for var. *vernalis* in the alternating light treatment (2.0 days) compared to complete darkness (2.8 days). In each of the other two temperature regimes, germination of both varieties commenced faster in complete darkness (3.0 days, in nearly all cases), than in alternating light (approximately 4.0 days, in all cases) (Tables 1 and 2). The slowest time to start germination for each variety did not vary significantly, either according to the magnitude or to temperature range, with var. *verbenaca* (4.2 days at 25/12°C) commencing just prior to var. *vernalis* (4.3 days at 20/15°C).

**Table 1:** Effect of temperature with photoperiod, osmotic potential, NaCl concentration, pH and seeding depth on germination or emergence parameters of *S*. *verbenaca* var. *verbenaca*.

Treatments		Time start to germination or emergence in days (SE)	T_50_ or E_50_ in days (SE)	MGT or MGT in days (SE)	GI or EI (SE)
Temperature °C	30/20 with 12 h light	3.0^b^ (0.0)	1.7^bc^ (0.45)	5.2^c^ (0.7)	4.1^bc^ (0.24)
	30/20 with 24 dark	3.0^b^ (0.0)	3.4^c^ (0.16)	4.0^d^ (0.09)	4.9^ab^ (0.20)
	20/15 with 12 h light	3.7^a^ (0.25)	6.4^a^ (0.15)	7.1^a^ (0.07)	2.7^d^ (0.08)
	20/15 with 24 dark	3.0^b^ (0.0)	3.6^c^ (0.55)	3.8^d^ (0.14)	5.1^a^ (0.30)
	25/12 with 12 h light	4.2^a^ (0.25)	5.6^ab^ (0.24)	6.0^b^ (0.24)	3.3^cd^ (0.13)
	25/12 with 24 dark	3.0^b^ (0.0)	3.4^c^ (0.01)	3.9^d^ (0.02)	4.9^ab^ (0.20)
	HSD at 0.05	0.64	1.43	0.74	0.92
Osmotic potential (MPa)	Control	3.0^b^ (0.0)	3.9^b^ (0.02)	4.4^b^ (0.02)	4.4^a^ (0.08)
	-0.05	3.5^b^ (0.29)	4.0^b^ (0.11)	4.5^b^ (0.11)	4.0^ab^ (0.06)
	-0.1	3.5^b^ (0.29)	3.8^b^ (0.24)	4.3^b^ (0.24)	3.45^b^ (0.38)
	-0.2	3.7^b^ (0.25)	4.0^b^ (0.24)	4.5^b^ (0.22)	1.8^c^ (0.15)
	-0.4	7.0^a^ (0.41)	6.5^a^ (0.42)	7.0^a^ (0.41)	0.1^d^ (0.01)
	-0.6	NG	NG	NG	NG
	HSD at 0.05	1.22	1.06	1.04	0.82
NaCl concentration (mM)	Control	3.0^d^ (0.0)	3.1^c^ (0.03)	3.6^d^ (0.02)	5.31^a^ (0.2)
	50	4.0^c^ (0.0)	4.5^c^ (0.22)	5.1^c^ (0.13)	3.8^b^ (0.15)
	100	5.0^b^ (0.0)	6.1^b^ (0.48)	6.6^b^ (0.41)	2.6^c^ (0.13)
	150	4.7^bc^ (0.25)	6.9^ab^ (0.52)	7.1^ab^ (0.28)	1.3^d^ (0.33)
	200	7.0^a^ (0.41)	7.6^a^ (0.24)	7.7^a^ (0.14)	0.23^e^ (0.04)
	250	NG	NG	NG	NG
	HSD at 0.05	0.93	1.51	1.03	0.74
pH	Control	3.0^a^ (0.0)	3.9^abc^ (0.02)	4.4^bc^ (0.02)	4.4^ab^ (0.08)
	5	3.0^a^ (0.0)	3.5^cd^ (0.11)	4.0^cd^ (0.12)	4.8^a^ (0.30)
	6	2.7^a^ (0.25)	2.9^d^ (0.25)	3.5^d^ (0.24)	5.2^a b^ (0.43)
	7	3.0^a^ (0.0)	3.5^bcd^ (0.04)	4.1^bcd^ (0.05)	5.0^a b^ (0.06)
	8	3.0^a^ (0.0)	4.3^ab^ (0.18)	4.7^ab^ (0.18)	4.1^ab^ (0.26)
	9	3.0^a^ (0.0)	3.6^bcd^ (0.06)	4.2^bc^ (0.11)	4.4a^b^ (0.04)
	10	3.0^a^ (0.29)	4.5^a^ (0.27)	5.1^a^ (0.22)	3.3^b^ (0.31)
	HSD at 0.05	0.43	0.75	0.71	1.17
Seeding depth (cm)	0	2.2^c^ (0.25)	3.3^d^ (0.24)	3.8^d^ (0.19)	5.6^a^ (0.43)
	0.5	4.5^b^ (0.29)	5.0^c^ (0.27)	5.7^c^ (0.25)	2.8^b^ (0.08)
	1.0	7.0^a^ (0.0)	6.9^b^ (0.17)	7.7^b^ (0.17)	1.7^c^ (0.11)
	2.0	7.5^a^ (0.05)	9.2^a^ (0.43)	9.7^a^ (0.10)	0.48^d^ (0.02)
	4.0	NE	NE	NE	NE
	HSD at 0.05	1.32	1.22	0.78	0.95

T_50_ or E_50,_ time to obtain 50% germination; MGT or MET, mean germination or emergence time; GI, germination index; EI, emergence index; NG or NE, no germination or emergence. The values within the column followed by different letters were significantly different at P ≤ 0.05. HSD = Tukey's honestly significant difference. SE = Standard error of the means.

**Table 2:** Effect of temperature with photoperiod, osmotic potential, NaCl concentration, pH and seeding depth on germination or emergence parameters of *S*. *verbenaca* var. *vernalis*.

Treatments		Time start to germination or emergence in days (SE)	T_50_ or E_50_ in days (SE)	MGT or MGT in days (SE)	GI or EI (SE)
Temperature °C	30/20 with 12 h light	2.0^c^ (0.0)	2.9b (0.30)	3.4b (0.30)	6.4a (0.55)
	30/20 with 24 dark	2.8^b^ (0.25)	3.0b (0.30)	3.8b (0.27))	5.6a (0.61)
	20/15 with 12 h light	4.3^a^ (0.25)	5.9a (0.31)	6.6a (0.32)	2.9b (0.10)
	20/15 with 24 dark	3.0^b^ (0.0)	3.2b (0.18)	3.9b (0.20)	5.3a (0.31)
	25/12 with 12 h light	4.0^a^ (0.0)	5.6a (0.27)	6.0a (0.26)	3.1b (0.09)
	25/12 with 24 dark	3.0^b^ (0.0)	3.4b (0.16)	3.9b (0.03)	5.0a (0.14)
	HSD at 0.05	0.65	1.12	1.13	1.65
Osmotic potential (MPa)	Control	2.0^c^ (0.0)	3.0^c^ (0.04)	3.4^c^ (0.04)	6.1^a^ (0.12)
	-0.05	2.0^c^ (0.0)	2.6^c^ (0.19)	3.1^c^ (0.14)	6.1^a^ (0.30)
	-0.1	3.0^b^ (0.0)	3.3^c^ (0.02)	3.8^c^ (0.02)	4.7^b^ (0.08)
	-0.2	4.0 ^a^ (0.0)	4.6^b^ (0.11)	5.3^b^ (0.13)	3.0^c^ (0.03)
	-0.4	4.5^a^ (0.28)	5.8^a^ (0.37)	6.8^a^ (0.52)	1.0^d^ (0.11)
	-0.6	NG	NG	NG	NG
	HSD at 0.05	0.56	0.85	1.09	0.69
NaCl concentration (mM)	Control	2.0^d^ (0.0)	2.6^c^ (0.23)	3.1^d^ (0.22)	6.7^a^ (0.45)
	50	3.0^cd^ (0.0)	3.3^c^ (0.04)	3.8^cd^ (0.04)	4.6^b^ (0.18)
	100	3.3^c^ (0.25)	3.8^bc^ (0.28)	4.3b^bc^ (0.28)	4.0^b^ (0.43)
	150	4.0^c^ (0.40)	4.6^b^ (0.26)	5.2^b^ (0.24)	2.6^c^ (0.23)
	200	5.5^b^ (0.28)	6.8^a^ (0.30)	7.3^a^ (0.22)	1.7^c^ (0.13)
	250	6.8^a^ (0.25)	7.0^a^ (0.40)	7.4^a^ (0.32)	0.3^d^ (0.27)
	HSD at 0.05	1.12	1.25	1.07	1.28
pH	Control	2.0^b^ (0.0)	2.3^ab^ (0.05)	3.3^cd^ (0.04)	6.1^ab^ (0.12)
	5	3.0^a^ (0.0)	3.5^a^ (0.02)	4.0^abc^ (0.03)	4.8^b^ (0.38)
	6	3.0^a^ (0.0)	3.4^ab^ (0.06)	3.9^abc^ (0.05)	5.1^b^ (0.12)
	7	2.0^a^ (0.0)	2.7^b^ (0.28)	3.2^d^ (0.27)	6.8^a^ (0.51)
	8	2.5^ab^ (0.28)	3.1^ab^ (0.27)	3.6^bcd^ (0.24)	55^ab^ (0.57)
	9	3.0^a^ (0.0)	3.5^a^ (0.02)	4.2^a^ (0.02)	6.6^b^ (0.16)
	10	3.0^a^ (0.0)	3.6^a^ (0.03)	4.2^a^ (0.04)	4.6^b^ (0.14)
	HSD at 0.05	0.50	0.68	0.65	0.55
Seeding depth (cm)	0	3.1^c^ (0.0)	4.3^c^ (0.13)	4.7^d^ (0.11)	4.3^a^ (0.13)
	0.5	4.5^b^ (0.28)	5.0^c^ (0.26)	5.6^c^ (0.27)	3.5^b^ (0.19)
	1.0	6.0^a^ (0.0)	6.6^b^ (0.05)	7.2^b^ (0.13)	2.1^c^ (0.14)
	2.0	6.7^a^ (0.48)	9.1^a^ (0.48)	9.5^a^ (0.26)	0.6^d^ (0.05)
	3.0	NE	NE	NE	NE
	4.0	NE	NE	NE	NE
	HSD at 0.05	1.17	1.18	0.88	0.57

T_50_ or E_50,_ time to obtain 50% germination; MGT or MET, mean germination or emergence time; GI, germination index; EI, emergence index; NG or NE, no germination or emergence. The values within the column followed by different letters were significantly different at P ≤ 0.05. HSD = Tukey's honestly significant difference. SE = Standard error of the means.

The shortest time to reach 50% of the maximum germination (*T*_*50*_) was observed in the 30/20°C temperature range and alternating light regime for var. *verbenaca* (1.7 days), and the lowest mean germination time (*MGT*) was observed in var. *vernalis* (3.4 days), under the same temperature and light conditions. In both varieties, the highest *T*_*50*_ and *MGT* were observed at 20/15°C in the alternating light regime, 6.4 (*T*_*50*_) and 7.1 (*MGT*) days for var. *verbenaca* and 5.9 (*T*_*50*_) and 6.6 (*MGT*) days for var. *vernalis*. An indication of germination not being inhibited by complete darkness in the two lower temperature ranges is given by the *T*_*50*_ and *MGT* values being significantly less in the complete darkness regime, compared to alternating light (Tables 1 and 2).

The germination index (*GI*), which is a measure of the percentage and speed of germination [25], indicates a slight difference between each variety in response to temperature and light regime (Tables 1 and 2). Higher values for this measure indicate a greater rate of germination [28]. Of these two varieties, var. *verbenaca* appears to favour lower temperatures than var. *vernalis*, and is also responsive to an absence of light, since the maximum *GI* for var. *verbenaca* (5.1) was observed at 20/15°C in the complete darkness regime. However, with the maximum *GI* of 6.4, and in addition to nearly all of the other *GI* values being higher (Tables 1 and 2), there is an overall indication of a stronger germination response in var. *vernalis* than var. *verbenaca*.

The observed trend, overall, for var. *verbenaca* indicates less variation according to temperature on the time to commence germination, compared to var. *vernalis*, since germination commenced in 3 days for each temperature range for at least one of the light treatments. By comparison, germination of var. *vernalis* took slightly longer overall, according to light treatment, for the two lower temperature ranges, but less time overall than var. *verbenaca* in the highest temperature range. By comparison with these two *Salvia verbenaca* varieties, some species are more dependent on light as a germination cue, e.g., *Galenia pubescens* [8] and *Chenopodium album* [12], whereas other species, e.g., *Asphodelus tenuifolius* are not significantly inhibited by a lack of light [13]. It is possible that the amount of light may also have an influence on the rate of germination [17], and whilst this phenomenon was not investigated in this study, it may be of interest for future research into these two *S*. *verbenaca* varieties.

The values of the parameters for the rate of germination over time, *T*_*50*_ and *MGT*, were slightly higher for var. *verbenaca* than var. *vernalis*, indicating that in a situation where both are present in the soil seed bank, var. *vernalis* may possibly germinate prior to var. *verbenaca*. Javaid and Tanveer (2014) [17] found a similar trend for two species of *Emex*, at relatively low temperatures (10°C to 20°C), with *T*_*50*_ and *MGT* of *E*. *spinosa* indicating a more rapid germination response than *E*. *australis*. However, at higher temperatures (20°C to 30°C) there was less difference between each species in the time to commence germination and its overall rate.

The *GI* value for var. *vernalis*, in addition to higher overall values of *GI*, indicates a higher and more rapid germination response than var. *verbenaca*. In particular, the higher value at the higher temperature range indicates a tolerance for comparatively higher temperatures. The germination total percentage and rate of *E*. *spinosa* germination was higher than that of *E*. *australis* at a higher temperature [17], which is a similar to the comparison of var. *vernalis* and var. *verbenaca*. Also, although being slightly less in absolute percentage germination compared to both *S*. *verbenaca* varieties, the germination of some members of the Lamiaceae family, *Salvia aegyptiaca* [29], *Hyssopus officinalis*, *Ocimum basilicum* and *Origanum vulgare* [30], as well as *Medicago ruthenica* [31], *G*. *pubescens* [8] and *C*. *album* (xeric population) [12] were broadly similar over the entire range of temperatures tested in this study. These similarities in the response of all these species are possibly due to similarity of climates in their regions of origin, where they are likely to experience dry weather at warm times of the year and wetter conditions during cooler times.

### Effect of osmotic potential

**Fig 2:**
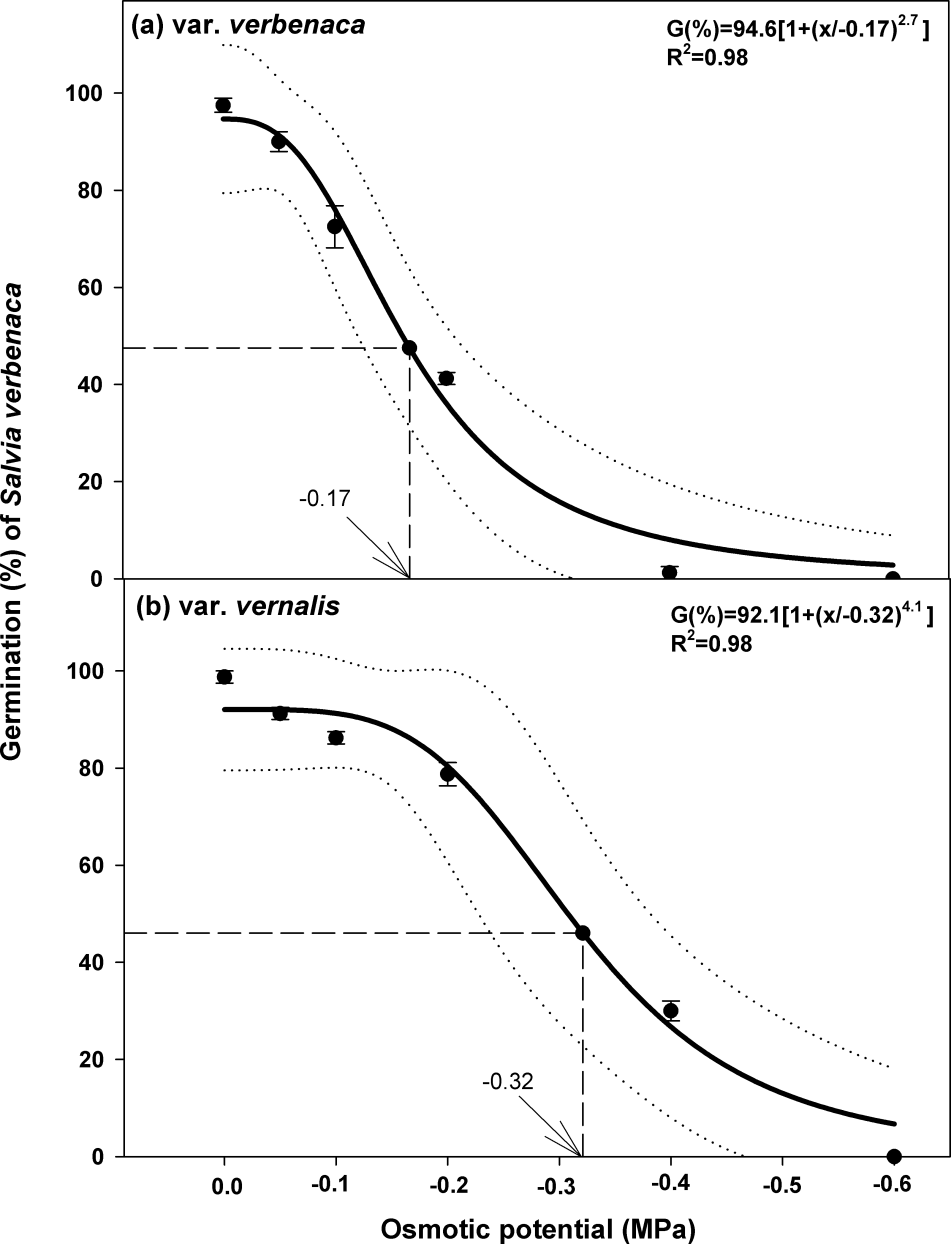
**Effect of osmotic potential on seed germination of *S*. *verbenaca* varieties (a) *verbenaca* and (b) *vernalis*.** Bold line represents a three-parameter logistic model fitted to the data. Vertical dash line represents X-axis value at 50% of the maximum germination. Vertical bars represent ± standard error of the mean.

Germination in the distilled water control was greater than 97% in both varieties (Fig 2A and 2B). However, germination of var. *verbenaca* decreased more rapidly with decreasing osmotic potential, compared to var. *vernalis*. At an osmotic potential of -0.4 MPa, germination of var. *verbenaca* and var. *vernalis* was 5% and 30%, respectively, although germination of both varieties was completely inhibited at an osmotic potential of -0.6 MPa. A three-parameter logistic model was fitted to the germination data of both varieties, which indicated that the maximum germination of var. *verbenaca* was reduced to 50% at only -0.17 MPa, whereas the maximum germination of var. *vernalis* was reduced to 50% at -0.32 MPa (Fig 2A and 2B). This indicates that seeds of var. *verbenaca* are less tolerant of lack of soil moisture than var. *vernalis*.

In both varieties, there was a trend of delayed germination with decreasing osmotic potential, as indicated by the overall trend of rising values for time to start germination, *T*_*50*_, and *MGT* (Tables 1 and 2). Also, there was variation in the response of each variety to an absence of, or relatively low, water stress. Seeds of var. *verbenaca* started germinating after 3 days with distilled water, but var. v*ernalis* started germinating after only 2 days in either distilled water or an osmotic potential solution of -0.05 MPa. The time to start germination was longest for both varieties in a solution of -0.4 MPa osmotic potential; 7 days for var. *verbenaca* and 4.5 days for var. *vernalis*. The *T*_*50*_ and *MGT* were also lowest for both varieties in the control, but higher for var. *verbenaca* (*T*_*50*_, 3.9 days; *MGT*, 4.4 days) than for var. *vernalis* (*T*_*50*_, 3.0 days; *MGT*, 3.4 days). These parameters were highest for both varieties at -0.4 MPa and values for var. *verbenaca* (*T*_*50*_, 6.5 days; *MGT*, 7.0 days) were higher than for var. *vernalis* (*T*_*50*_, 5.8 days; *MGT*, 6.8 days). For var. *vernalis*, *GI* was highest in both of the distilled water control and -0.05 MPa solution (6.1 for both), and for var. *verbenaca GI* was highest in the distilled water control (4.4). For both varieties, *GI* was lowest in the -0.4 MPa osmotic potential solution, 0.1 for var. *verbenaca* and 1.0 for var. *vernalis*.

Each species reacted slightly differently to increasing moisture stress, with var. *verbenaca* displaying less adaptation to a lack of soil moisture compared to var. *vernalis*. This is supported by the observation that var. *verbenaca* germinated less overall and more slowly than var. *vernalis*, as indicated by the more rapid drop off in germination (Fig 2A and 2B) and lower figures for *GI* (Tables 1 and 2) with increasing moisture stress, compared to var. *vernalis*. The pattern of reduction in germination rate between the two *Salvia verbenaca* varieties was similar to that between *E*. *spinosa* and *E*. *australis*, with a more rapid decline of germination observed in the latter than the former, although both were more resistant to moisture stress than both of the *S*. *verbenaca* varieties. The maximum germination rate of *E*. *spinosa* was not reduced to 50% until -0.33 MPa, very similar to var. *vernalis*, but *E*. *australis* was slightly more resistant to an increase of osmotic potential than var. *verbenaca*, being reduced to 50% of the maximum germination at -0.26 MPa (compared to -0.17 for var. *verbenaca*). Other species that respond similarly to var. *vernalis* include *G*. *pubescens* (no significant reduction before -0.2 MPa) [8] and *A*. *tenuifolius* (rapid reduction after -0.2 MPa, 50% by -0.38 MPa) [13]. By contrast, *Trianthema portulacastrum* (horse purslane) was less affected by moisture stress, with a 50% reduction in maximum germination at -0.50 MPa [6]. This indicates that in a situation of competition between these weeds, *Trianthema portulacastrum* would be the least affected by lack of moisture than all the other species, but *S*. *verbenaca* var. *vernalis* would be more likely to germinate than var. *verbenaca* at low soil moisture levels.

### Effect of salt stress

**Fig 3:**
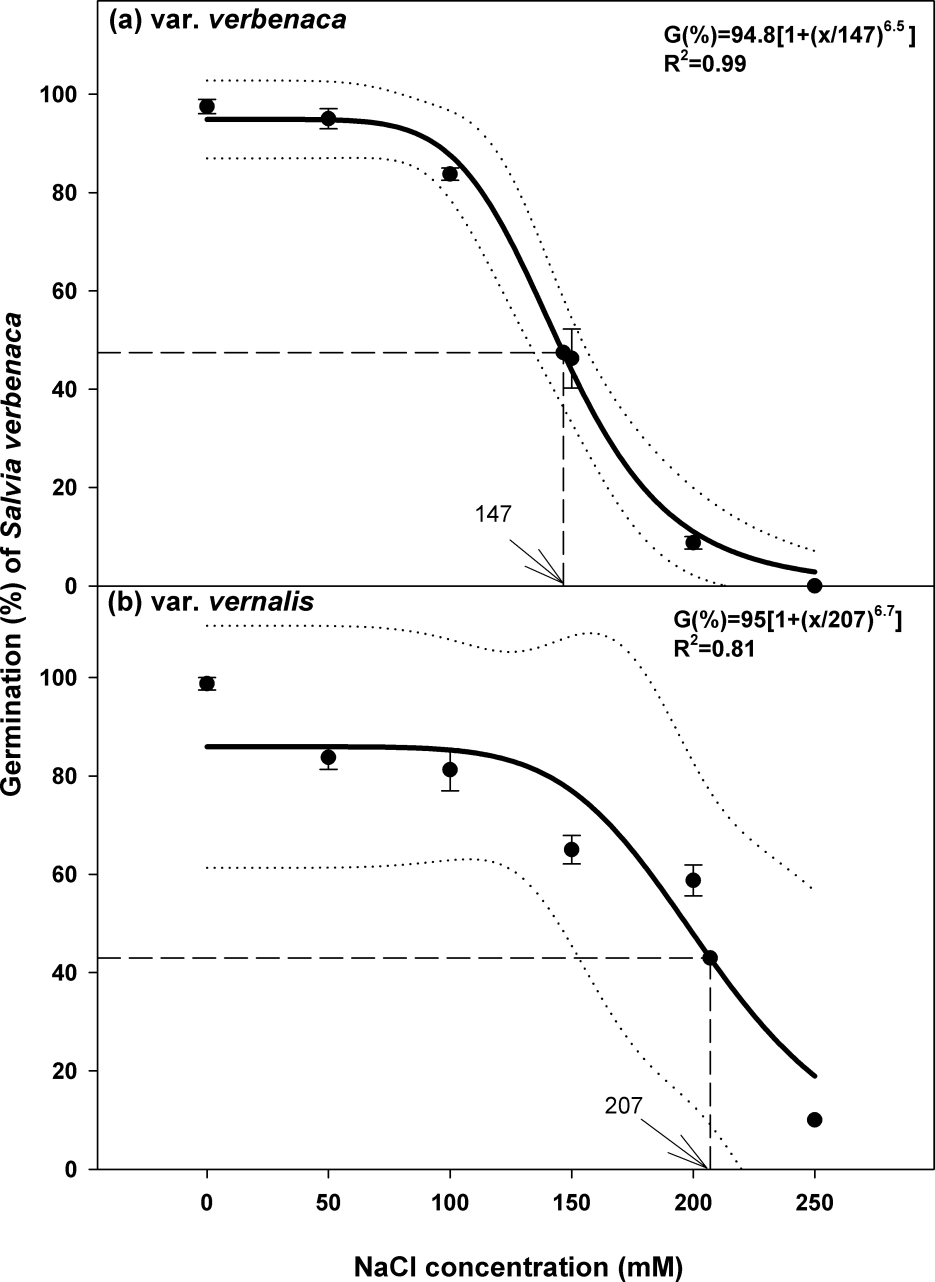
**Effect of NaCl concentration on seed germination of *S*. *verbenaca* varieties (a) *verbenaca* and (b) *vernalis*.** Bold line represents a three-parameter logistic model fitted to the data. Vertical dash line represents X-axis value at 50% of the maximum germination. Vertical bars represent ± standard error of the mean.

To test the effect of salt stress, a three-parameter logistic model was fitted to the seed germination data for each variety (Fig 3A and 3B). This model estimated that the highest germination was 95% in the control (no salt stress), and seed germination decreased in both varieties with increasing salt concentration. Germination of var. *verbenaca* in the 50 mM solution was only slightly less than the control (92%), but var. v*ernalis* was affected more strongly, with germination reduced to less than 85% in this concentration of NaCl. However, germination of var. *vernalis* was less affected at higher concentrations than var. *verbenaca*, in which germination declined significantly above 100 mM NaCl. Germination of var. *verbenaca* was reduced to 50% of the maximum germination at 147 mM (Fig 3A), contrasting with 207 mM for var. *vernalis* (Fig 3B). Germination of var. *verbenaca* was extinguished at 250 mM, but was still approximately 10% in var. *vernalis* at this concentration. In spite of the apparent sensitivity to low saline concentrations, there is an indication that var. *vernalis* has more ability to tolerate moderate to high saline conditions than var. *verbenaca*.

Overall, germination was slower with increasing saline concentration for var. *verbenaca* compared to var. *vernalis*. The time to start germination of var. *verbenaca* was delayed by one day in the distilled water control (3.0 days) and 50 mM NaCl solution (4.0 days), and more than one day at 100 mM (5.0 days), compared to var. *vernalis* (distilled water, 2.0 days; 50 mM, 3.0 days; 100 mM, 3.3 days) (Tables 1 and 2). At a concentration of 150 mM commencement of germination was delayed less than one day between each variety (var. *verbenaca*, 4.7 days; var. *vernalis*, 4.0 days) and less than two days at 200 mM (var. *verbenaca*, 7.0 days; var. *vernalis*, 5.5 days). There was no germination of var. *verbenaca* at 250 mM, and germination did not commence in var. *vernalis* until nearly one week (6.8 days).

The parameters *T*_*50*_ and *MGT* were higher overall in var. *verbenaca* than that in var. *vernalis* in each concentration of NaCl (Tables 1 and 2), indicating slower germination of var. *verbenaca* in response to increasing salinity compared to var. *vernalis*. For the percentage and rate of germination, *GI* was lower for var. *verbenaca*, with a maximum of 5.31 in the distilled water control, compared to a maximum of 6.7 in the same treatment for var. *vernalis*. This indicates that germination occurs most rapidly and at the highest rate in the distilled water control in both varieties, but that var. *vernalis* germinates more rapidly and is more resistant to salinity than var. *verbenaca*.

The differential delay in germination according to variety, with germination of var. *vernalis* being less affected at the highest rate of salinity overall, indicates that some adaptation to the saline conditions is present in this variety. Its ability to germinate slightly earlier and at a slightly higher rate, indicate that this variety is more likely to establish in moderate to highly saline conditions, compared to var. *verbenaca*. Weeds that may germinate in similar saline conditions include *Salvia aegyptiaca* [29], *Medicago ruthenica* [31], *G*. *pubescens* [8], *C*. *album* [12], *E*. *spinosa* [17], and *T*. *portulacastrum* [6], but not *A*. *tenuifolius* [13] or *E*. *australis* [17], in which the maximum germination is reduced to 50% at 43 and 91 mM, respectively, compared to more than 100 mM for all of the other species. As a result, in more saline regions the establishment of the last two species is less favoured than either of the *Salvia verbenaca* varieties or any of the other four species.

### Effect of pH

**Fig 4:**
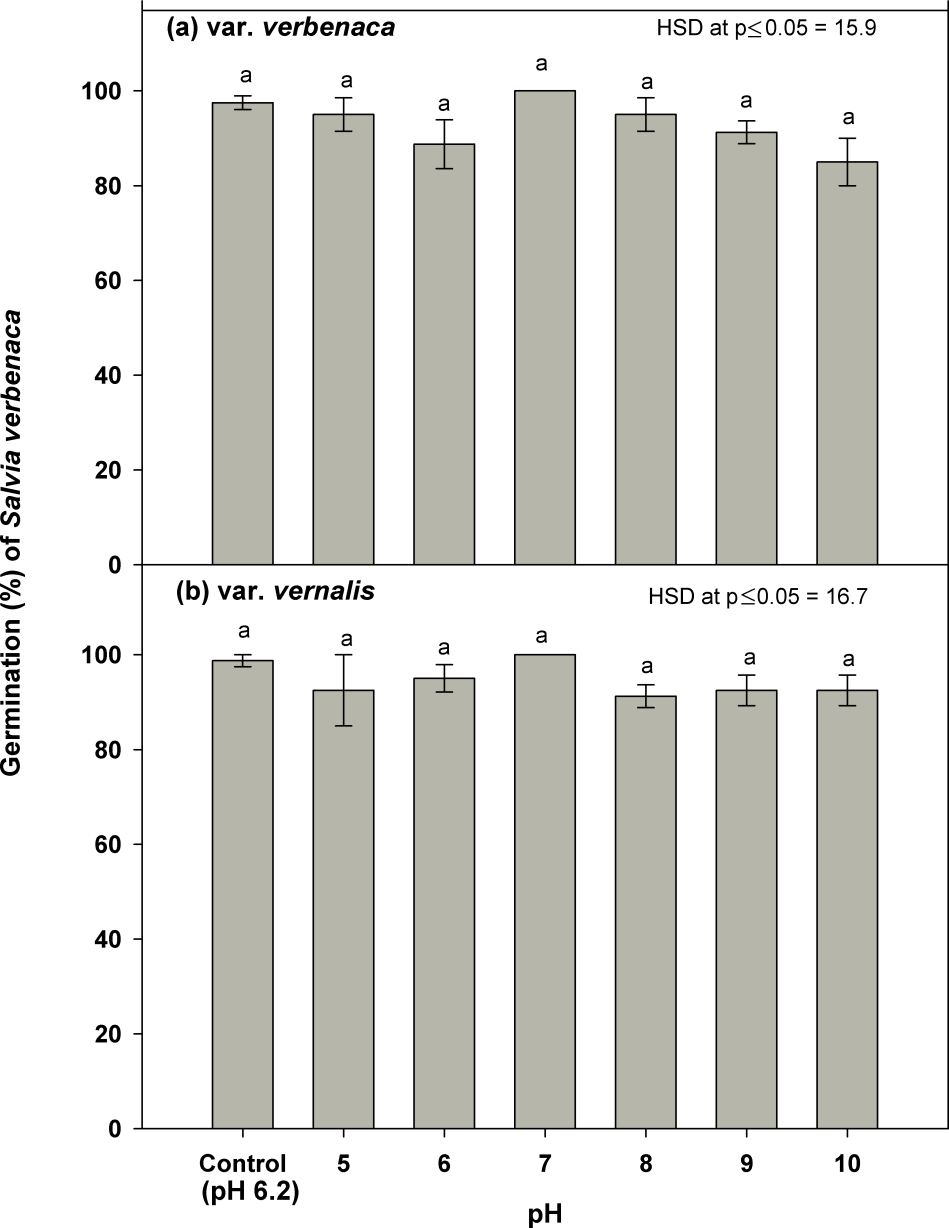
**Effect of pH on germination of *S*. *verbenaca* varieties (a) *verbenaca* and (b) *vernalis*.** Nails on the vertical bars represent standard error of the means.

Germination of both varieties was strong to very strong (not less than 85%) in all buffer solutions from pH 5 to 10, and in the control (pH 6.2) (Fig 4A and 4B). The highest germination was observed in the pH 7 buffer and control solution, 100% and 97% respectively, for both varieties. In var. *verbenaca*, germination in the pH 5 and 8 buffers was most similar to the control treatment. However, germination was slightly reduced, to 90% and 85% respectively, in the pH 9 and 10 buffers (Fig 4A). For var. *vernalis*, there was less variation in germination overall, with a minimum of approximately 90% in the pH 8 buffer and slightly more than this percentage in the pH 5, 6, 9 and 10 buffers (Fig 4B).

None of the parameters for the rate of germination varied significantly. Time to first germination occurred earliest for var. *vernalis* in the control (pH 6.2) (2.0 days), pH 7 (2.0 days) and pH 8 (2.5 days) (Tables 1 and 2), and germination commenced in each of the remaining buffers, pH 5, 6, 9 and 10, at 3 days. Commencement of germination in var. *verbenaca* was very slightly slower, occurring first in pH 6 (2.7 days), and subsequently in the control (pH 6.2), pH 5, 7, 8, 9 and 10 buffers at 3 days. The minimum (2.9 days) and maximum (4.5 days) *T*_*50*_ was observed in var. *verbenaca* at pH 6 and 10, respectively, as was the minimum (3.5 days) and maximum (4.5 days) *MGT*. In var. *vernalis* minimum and maximum was 2.3 and 3.6 at control and pH 10, respectively. However, the minimum and maximum *MGT* did not follow exactly the same pattern as var. *verbenaca*. The minimum MGT for var. *vernalis* occurred at pH 7 (3.2 days), while the maximum (4.2 days) was observed at pH 9 and 10. The maximum *GI* for each variety varied according to pH, with var. *verbenaca* responding more strongly to relatively more acidic conditions (5.3 days at pH 6), compared to var. *vernalis* (6.8 days at pH 7), and each responding least in the same, alkaline, pH of 10 (var. *verbenaca* 3.3 days; var. *vernalis* 4.6 days). The minimum *GI* for both varieties was observed at pH 10, 3.3 days for var. *verbenaca* and 4.6 days for var. *vernalis*.

Since the overall germination response was very strong, and each of these varieties did not vary significantly in their response to varying pH, there is insufficient evidence to conclude that variation in soil pH across geographic regions is likely to restrict the distribution of either variety. Therefore, alteration of soil pH, for example by the addition of lime, is unlikely to prevent the occurrence of either variety in agricultural regions. Additionally, these varieties can be expected to occur in a wide variety of soil types in either modified or natural ecosystems.

These findings are similar to those found for *T*. *portulacastrum*, *A*. *tenuifolius*, *E*. *spinosa*, [6], [13], [17], but contrast slightly with *G*. *pubescens* and *E*. *australis* [8], [17]. In the first three species, response was very similar to both *S*. *verbenaca* varieties, since the germination rates were consistently high in response to change in pH from low values to high. By contrast, *G*. *pubescens* indicated less preference for neutral pH and more for either acid or alkaline conditions [8], contrasting with *S*. *verbenaca*, in which the highest germination was observed at a neutral pH (7). Maximum germination of *E*. *australis* was also at pH 7, but there was a marked decline in germination rate above pH 7.5, indicating less preference for alkaline conditions, compared to *S*. *verbenaca*.

### Effect of burial depth

**Fig 5:**
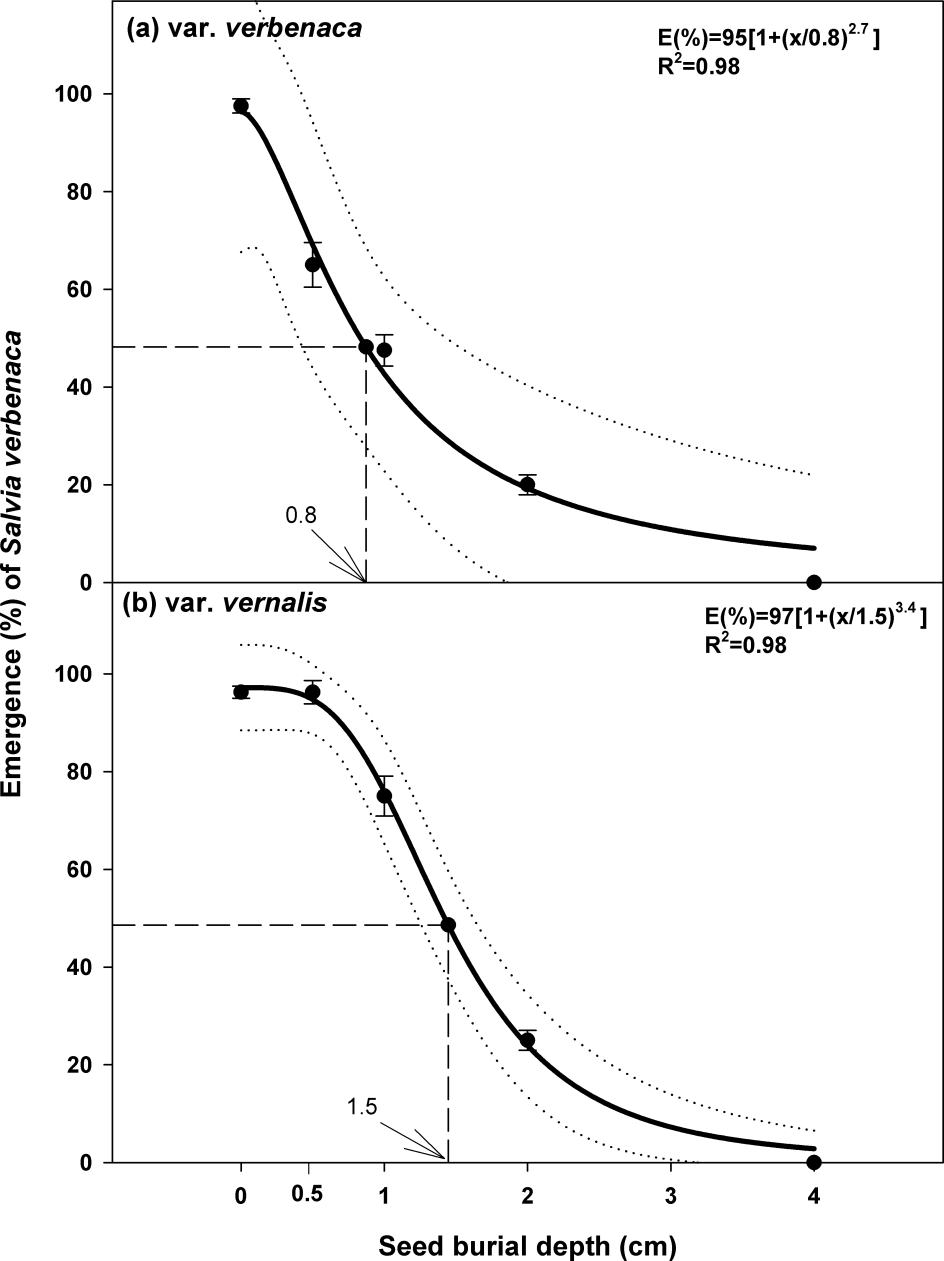
**Effect of seeding depth on seed germination/emergence of *S*. *verbenaca* varieties (a) *verbenaca* and (b) *vernalis*.** Bold line represents a three-parameter logistic model fitted to the data. Vertical dash line represents X-axis value at 50% of the maximum germination. Vertical bars represent ± standard error of the mean.

A three-parameter logistic model was fitted to the seed germination and seedling emergence data (Fig 5A and 5B). Seeds of var. *verbenaca* placed at the soil surface (0 cm) showed 97% germination. Subsequently, emergence decreased progressively with increasing depth of burial and was completely inhibited at 4 cm. The model estimated that burial depth for 50% inhibition of the maximum seedling emergence was 0.8 cm (Fig 5A). By contrast, germination of var. *vernalis* was 97% for seeds placed on the soil surface, but this percentage was not reduced at all in seeds buried at 0.5 cm (Fig 5B). Subsequently, emergence progressively decreased as depth increased. As was the case with var. *verbenaca*, emergence was completely inhibited at burial depth of 4 cm. The fitted model estimated that 50% reduction of the maximum seedling emergence occurred at 1.5 cm burial depth. Shallow burial, therefore, had less effect on the emergence of var. *vernalis* than on var. *verbenaca*.

Seedling germination and emergence, as indicated by time to first emergence, time to 50% of the maximum emergence (*E*_*50*_) and mean emergence time (*MET*), all increased with burial depth for both varieties (Tables 1 and 2). For the control (0 cm), first emergence of var. *verbenaca* occurred prior to var. *vernalis*, at 2.2 days and 3.1 days after sowing, respectively. However, the subsequent increase in burial depth, of up to 2 cm, progressively delayed the seedling emergence of var. *verbenaca* (0.5 cm, 4.5 days; 1 cm, 7.0 days; 2 cm, 7.5 days), compared to var. *vernalis* (0.5 cm, 4.5 days; 1 cm, 6 days; 2 cm, 6.7 days). No germination occurred for either variety of seeds buried at 4 cm. The *E*_*50*_ and *MET* also increased progressively with increasing depth of burial in both varieties, with the minimums at 0 cm (*E*_*50*_ and *MET*: 3.3 and 3.8 days, var. *verbenaca*; 4.3 and 4.7 days, var. *vernalis*) maximums at 2 cm (*E*_*50*_ and *MET*: 9.2 and 9.7 days, var. *verbenaca*; 9.1 and 9.5 days, var. *vernalis*). Emergence index (*EI*) decreased in both varieties with increasing burial depth. For var. *verbenaca* seeds placed on the soil surface, *EI* was 5.6, and this progressively decreased to 0.48 at 2 cm. Similarly, for var. *vernalis* the *EI* at 0 cm was 4.3 and this decreased to 0.6 *EI* at 2 cm. No emergence was recorded at 4 cm burial depth in either variety. These figures for *EI* indicate that at the soil surface, var. *verbenaca* germinates more readily and faster than var. *vernalis;* however this trend is reversed with increasing burial depth.

Each of these varieties did not vary significantly in their response to being placed on the soil surface. However, var. *vernalis* seed germinated more frequently and rapidly than var. *verbenaca* on the surface and at the shallowest burial depth, perhaps indicating a degree of resilience to shallow burial, including slightly less sensitivity to the absence of light, than the latter variety. Similarly to var. *verbenaca*, *G*. *pubescens* germinations rapidly reduced with increasing burial depth, reaching 50% of the maximum at less than 0.5 cm [8]. *Chenopodium album* germination response varied slightly by population, with both mesic and xeric germinating most strongly at the surface (0 cm), but after a longer time than either *Salvia* variety, (6 days) [12]. Additionally, germination of *C*. *album* ceased at a shallower depth than var. *vernalis*, although the mesic population of *C*. *album* continued to germinate more strongly from depths greater than 0.5 cm, in contrast to the xeric population. This is perhaps due to a larger seed size in the mesic population. By contrast to the cessation of germination with very shallow burial depths, *A*. *tenuifolius*, *T*. *portulacastrum* and both *Emex* species germinated at depths ranging from 2.7 to 9.7 cm. This is possibly due to variation seed size of these species, since the seeds of both *Emex* species are somewhat larger than *A*. *tenuifolius* and *T*. *portulacastrum* [32], [33], [34], [35]. However, the findings of these studies contrast with the findings for the current study, since germination of the smaller var. *vernalis* seeds (1.75 ± 0.014 mg) were not inhibited by burial depth than the larger var. *verbenaca* seeds (2.40 ± 0.045 mg).

The effect of seed size on germination according to population source of seed within a species was investigated by Capon and Brecht (1970) [36], with a finding that a rough correlation exists between seed size and total percent germination. We suggest that this phenomenon be explored for *S*. *verbenaca* var. *verbenaca* and var. *vernalis*, in order to establish if there is any variation in seed size and if this can go some way towards explaining the different germination responses such as the rate of germination and burial depth response in these two varieties. Additionally, Armengot and colleagues (2016) [37] found that tillage has a stronger inhibitory effect on perennial weed species, compared to annuals, therefore supporting further investigation of the effect of tillage on *S*. *verbenaca*.

## Conclusion

Each variety did not contrast significantly in their response to light, however var. *vernalis* appeared to be better adapted to higher temperatures, lower water availability and higher salinity than var. *verbenaca*, since it germinated more rapidly and at a slightly greater percentage, compared to var. *verbenaca*. Additionally, var. *vernalis* was able to germinate from a deeper burial depth, despite possessing a significantly smaller seed weight. Therefore, in a situation where both species are present in the soil seed bank, var. *vernalis* is likely to be more aggressive than var. *verbenaca* in stressful environmental conditions of temperature, water availability and moderate to high salinity. Since both germinated strongly in either periodic light or complete darkness, simply shading the soil if seeds are present on the surface would do little to inhibit the germination of either species. However, since burial of seeds of 2 cm or more is likely to inhibit both species from germination, we recommend that soil tillage be applied for the control of these species in an agricultural setting. In addition to the previous recommendation to further examine the influence of seed size from each variety on germination ability under different conditions, the effect of fire on seed germination of these species was not examined in this study; therefore we recommend this be investigated also.

## Supporting information

S1 File Data used in graph on excel sheet. (XLSX)
